# Recent advances in the *CRANK* software suite for experimental phasing

**DOI:** 10.1107/S0907444910052224

**Published:** 2011-03-18

**Authors:** Navraj S. Pannu, Willem-Jan Waterreus, Pavol Skubák, Irakli Sikharulidze, Jan Pieter Abrahams, Rudolf A. G. de Graaff

**Affiliations:** a Biophysical Structural Chemistry, Leiden University, PO Box 9502, 2300 RA Leiden, The Netherlands

**Keywords:** *CRANK*, experimental phasing

## Abstract

For its first release in 2004, *CRANK* was shown to effectively detect and phase anomalous scatterers from single-wavelength anomalous diffraction data. Since then, *CRANK* has been significantly improved and many more structures can be built automatically with single- or multiple-wavelength anomalous diffraction or single isomorphous replacement with anomalous scattering data. Here, the new algorithms that have been developed that have led to these substantial improvements are discussed and *CRANK*’s performance on over 100 real data sets is shown. The latest version of *CRANK* is freely available for download at http://www.bfsc.leidenuniv.nl/software/crank/ and from *CCP*4 (http://www.ccp4.ac.uk/).

## Abstract 2

Recent developments in the *CRANK* software suite for experimental phasing have led to many more structures being built automatically.

## 1. Introduction

**Figure 1:**
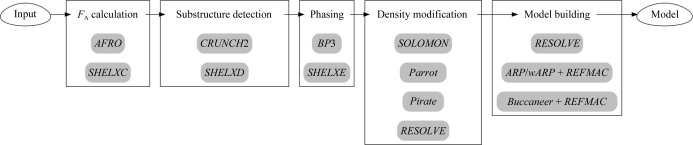
Flowchart showing the programs that *CRANK* can use and the steps that it can perform.

Currently, many software packages are available to automatically solve structures. The main aim of *CRANK* is to provide a user-friendly and automated system incorporating the latest computational developments in all stages of structure solution by experimental phasing. *CRANK* is not a monolithic system: users can define pipelines from a choice of many different programs. Fig. 1 ▶ shows the current steps that *CRANK* can perform and the programs that users can select to perform the task. The externally developed programs that *CRANK* can interface with are *SHELXC* (Sheldrick, 2008 ▶), *SHELXD* (Schneider & Sheldrick, 2002 ▶), *SHELXE* (Sheldrick, 2002 ▶), *DM* (Cowtan, 1994 ▶), *Parrot* (Cowtan, 2010 ▶), *Pirate* (Cowtan, 2000 ▶), *Buccaneer* (Cowtan, 2006 ▶) and *ARP*/*wARP* (Langer *et al.*, 2008 ▶), the latter two of which both iterate with *REFMAC* (Murshudov *et al.*, 2011 ▶), and *RESOLVE* (Terwilliger, 2000 ▶).

We are the main authors of the programs *AFRO* (Pannu *et al.*, in preparation) for *F*_A_ calculation, *CRUNCH*2 (de Graaff *et al.*, 2001 ▶) for substructure detection, *BP*3 (Pannu & Read, 2004 ▶) for substructure phasing, *SOLOMON* (Abrahams & Leslie, 1996 ▶) for density modification and *MULTICOMB* (Skubák, Waterreus *et al.*, 2010 ▶) for phase combination and are co-­authors of the program *REFMAC* (Murshudov *et al.*, 2011 ▶). These programs use multivariate maximum-likelihood methods that allow the observed diffraction data and any current models to be considered simultaneously at any stage in the structure-solution process. Thus, the wealth of information contained in the observed diffraction data can be used directly throughout the structure-solution process and not approximated or ignored as current approaches do after constructing an initial electron-density map.

Below, we provide a brief intuitive description of the novel methods in various steps in experimental phasing that we have developed since our first publication on *CRANK* (Ness *et al.*, 2004 ▶). We show the power of combining all of these new methods on over 100 real single-wavelength anomalous diffraction (SAD), multiple-wavelength anomalous diffraction (MAD) and single isomorphous replacement with anomalous scattering (SIRAS) data sets run automatically with minimal user input in *CRANK*.

The programs and methods we develop are not only available in *CRANK*, but also in *AutoRickshaw* (Panjikar *et al.*, 2005 ▶) and *ARP*/*wARP*. Furthermore, the original methods that we have developed have also been rewritten in mathematically identical forms in both *phenix.refine* and *Phaser* (Adams *et al.*, 2010 ▶).

## 2. Recent developments in *CRANK*

### 2.1. Substructure determination

After the diffraction data have been indexed and merged, *F*_A_ values are calculated for input to substructure-detection programs. |*F*_A_| values are the amplitudes of structure factors corresponding to the heavy atoms to be located. For SAD data, most programs use the absolute value of Bijvoet differences, Δ*F* = 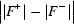, as an estimate of |*F*_A_|. Burla *et al.* (2002 ▶) proposed employing multivariate joint probability distributions to obtain the expected value for |*F*_A_| in an equation that contains three integrals. In order to obtain an analytical solution to the integrals, Burla *et al.* (2002 ▶) assume that the ‘Bijvoet phases’ are equal. We have obtained an expression requiring only one numerical integration without making this assumption. This approach has been implemented in the program *AFRO* and performs satisfactorily. Details of the implementation and test results will be given elsewhere (Pannu *et al.*, in preparation). The development version of *AFRO* containing the multivariate |*F*_A_| calculation is available in the latest version of *CRANK* and can be used as input for either *CRUNCH*2 or *SHELXD*.

Within *CRANK*, methods exist to validate whether a correct substructure has been determined and to terminate the substructure-detection step early. If a threshold value for a statistic used by the substructure-detection program has been reached or if a significant deviation exists between the best and worst score in different trials, the substructure-detection program will successfully terminate before running all trials. *CRANK* also provides an alternate and independent assessment of whether a correct substructure solution has been located: an option exists to run the substructure-phasing program *BP*3 quickly in ‘check’ mode and examine likelihood-based statistics to determine whether a correct and com­plete substructure has been found. The statistic that *CRANK* uses is a Luzzati parameter (Luzzati, 1952 ▶): if the average Luzzati parameter is greater than a threshold value (the default is 0.7) it is assumed that the full substructure has been found and substructure detection is terminated. Using likelihood methods to validate substructure detection has been available in *CRANK* for over three years (Pannu *et al.*, 2007 ▶) and this approach has been appreciated by *PHENIX* developers, who recently adopted it in their own suite (Paul Adams, CCP4 bulletin board, 31 July 2010).

### 2.2. Substructure phasing

To incorporate anomalous phase information, heavy-atom refinement programs such as *SHARP* (Bricogne *et al.*, 2003 ▶) or *MLPHARE* (Otwinowski, 1991 ▶; Collaborative Computational Project, Number 4, 1994 ▶) use a Gaussian function on observed Bijvoet differences (Δ*F* = |*F* ^+^| − |*F* ^−^|) centred on the ‘calculated’ Bijvoet difference that is determined from an assumed value of the ‘true’ structure factor and the heavy-atom structure factor (North, 1965 ▶; Matthews, 1966 ▶). Since, in general, the ‘true’ structure factor is not known for a SAD or MAD experiment, *SHARP* integrates out the amplitude and phase of the true structure factor. Furthermore, the estimate of measurement error for Bijvoet differences is determined by merging the measurement errors for Friedel pairs [σ_Δ*F*_ = 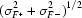], leading to suboptimal use of experimental information.

To input the observed structure factors directly, it is necessary to consider a joint probability of all observations given a current model. We have previously shown that this method provides better results compared with other approaches for the case of SAD (Pannu & Read, 2004 ▶; Ness *et al.*, 2004 ▶) as implemented in *BP*3. We have recently shown that better results may be obtained by deriving a multivariate function for SIRAS (Skubák *et al.*, 2009 ▶), which will be released in the next version of *CRANK*.

### 2.3. Density modification

In the density-modification procedure, the density-modified map is iteratively combined with the initial map obtained from experimental phasing. Current methods assume that these two maps are independent and propagate the initial map’s phase information indirectly through Hendrickson–Lattman co­efficients (Hendrickson & Lattman, 1970 ▶). We have applied a multivariate analysis that considers the observed Friedel pairs directly for a SAD experiment, accounts for the correlation between the initial and density-modified maps and refines the errors that can occur in a single-wavelength anomalous diffraction experiment. Results on many test cases show a significant improvement over the current state of the art (Skubák, Waterreus *et al.*, 2010 ▶): the maps produced by the multivariate phase-combination algorithm lead to many more structures being built automatically.

Despite the improvements in the quality of electron-density maps, the figures of merit remained escalated after density modification. To obtain more accurate figures of merit, we have recently developed and implemented a new cross-validated scheme for accurate error-parameter estimation in likelihood-based phase combination. This method leads to more reliable phase probability statistics from density modification and results in a further improvement in subsequent model building. In addition, the more accurate figures of merit enable a more reliable hand determination or identification of incorrect NCS operators used in density modification (Skubák & Pannu, 2011 ▶). These developments have been implemented in a new phase-combination program called *MULTICOMB* and can be used in conjunction with either *SOLOMON* or *Parrot*.

### 2.4. Automated model building and refinement

The incorporation of experimental phase information has previously been shown to improve refinement (Pannu *et al.*, 1998 ▶). However, the likelihood function developed, typically denoted MLHL, propagates the external phase information *via* Hendrickson–Lattman coefficients. Thus, the MLHL function is dependent on the accuracy and reliability of the coefficients that are input. Furthermore, in its derivation the MLHL function assumes that the experimental phase information (represented by Hendrickson–Lattman coefficients) is independent of the calculated structure factor. This assumption is questionable, as the experimental phase information is used to build an initial model. To overcome these issues, we considered and derived a multivariate likelihood function for SAD (Skubák *et al.*, 2004 ▶, 2005 ▶) and SIRAS (Skubák *et al.*, 2009 ▶) experiments. The likelihood functions take as input the diffraction data directly, the heavy-atom coordinates and the calculated structure factors and account for correlation between them. Compared with the other likelihood functions in *REFMAC*, more models are built automatically in *ARP*/*wARP* with the multivariate functions. The SAD and SIRAS functions in *REFMAC* are available in *CRANK* in model building with both *ARP*/*wARP* and *Buccaneer*.

### 2.5. Integration of programs and steps

**Figure 2:**
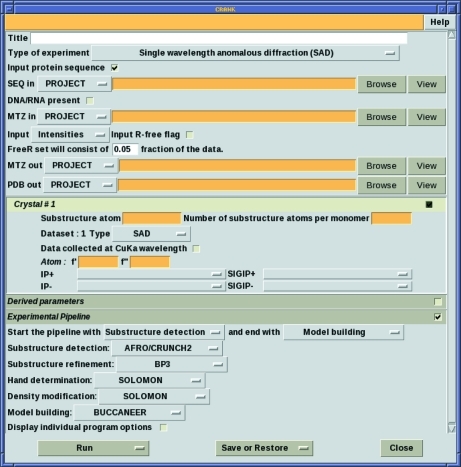
Screen shot of the *ccp*4*i* GUI for *CRANK*.

To support the integration of the different programs that it interfaces with, *CRANK* has a plug-in architecture and communicates between plug-ins *via* an XML file. At the moment, there are two methods available to generate an XML file that *CRANK* uses to run a pipeline: the program *GCX* and the *ccp*4*i* graphical user interface. Both interfaces to *CRANK* can be run with only minimal input: an MTZ file with the relevant column labels specified, a sequence file and the name, expected number and *f*′ and *f*′′ values for the heavy atoms. However, users can customize the settings for individual programs, define custom-made pipelines using any programs at each step and define the start and end step for a particular pipeline. Fig. 2 ▶ shows the *ccp*4*i* graphical user interface with its few required fields.

The program* GCX* allows *CRANK* to be run from a command line with a simple Unix script: more information on this can be obtained from the program’s documentation (http://www.ccp4.ac.uk/html/gcx.html). The test cases that are described below were run with *GCX*. Most users are likely to run *CRANK via* the *ccp*4*i* interface. The most convenient way to view a *CRANK* logfile is *via* the *Baubles* system, which can be initiated with the ‘View Annotated Logfile in a Web Browser’ option in *ccp*4*i*. Documentation for *CRANK* can be found at the the *CCP*4 wiki (http://www.ccp4wiki.org/), which includes information on how to best interpret the log files.

## 3. Methods

Here, we test the new methods described above on a wide range of real SAD, MAD and SIRAS merged diffraction data sets. For our tests, only the intensities or structure-factor amplitudes, along with the sequence for a protein monomer, the number of substructure atoms expected per monomer and the *f*′ and *f*′′ values for the substructure atoms were input. *CRANK* used *AFRO* and *CRUNCH*2 for substructure detection, *BP*3 for substructure phasing and *SOLOMON* with *MULTICOMB* for density modification. Three cycles of *Buccaneer* iterated with *REFMAC* were used for automated model building with iterative refinement. The default options or parameters were used in all programs. The defaults set by *CRANK* depend upon the particular experiment: for SAD data, *AFRO* uses the multivariate |*F*_A_| value calculation and *MULTICOMB* uses the multivariate SAD function for phase combination in density modification, while *Buccaneer* uses the SAD function implemented in *REFMAC*. For SIRAS data, *AFRO* calculates |*F*_A_| from either the anomalous signal or using isomorphous differences by determining which signal is greater. *BP*3 uses the uncorrelated SIRAS function described previously (Pannu *et al.*, 2003 ▶) and *SOLOMON* uses MLHL phase combination in *MULTICOMB*, while *Buccaneer* uses the multivariate SIRAS function in *REFMAC*. Finally, for MAD data *AFRO* chooses the wavelength with the greatest anomalous signal and calculates multivariate *F*_A_ values from it. Similar to SIRAS data, *SOLOMON* uses MLHL phase combination in *MULTICOMB* to perform density modification and *Buccaneer* uses the MLHL likelihood function in *REFMAC* for model refinement.

In the test cases below, the previous version of *CRANK*, version 1.3, is tested with the current version, version 1.4. The main differences between the two versions are the development version of *AFRO* that calculates multivariate |*F*_A_| values given SAD data and the use of *MULTICOMB* for phase combination in density modification, which were both introduced in version 1.4.

In total, we report results from 116 real data sets from several different sources listed in Appendix *A* [App appa]. The data sets cover a wide range of resolutions (from 0.94 to 3.29 Å) and anomalous scatterers, including selenium, sulfur, chloride, sulfate, manganese, bromide, calcium and zinc. Of the 116 data sets, 63 are MAD data sets, 46 are SAD data sets and seven are SIRAS data sets.

## 4. Results and discussion

**Figure 3:**
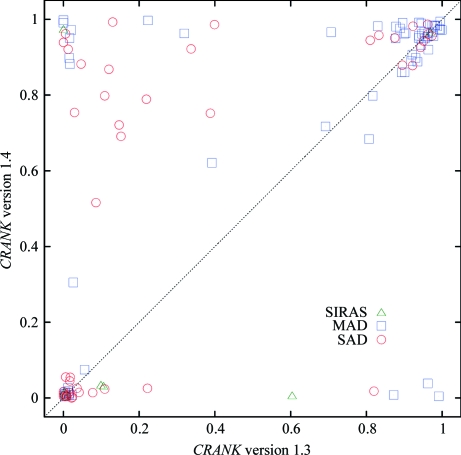
Graph of the fraction of the model automatically built with *CRANK* version 1.3 *versus CRANK* version 1.4. MAD data sets are shown as blue squares, SAD data sets are shown as red circles and SIRAS data sets are shown as green triangles.

**Figure 4:**
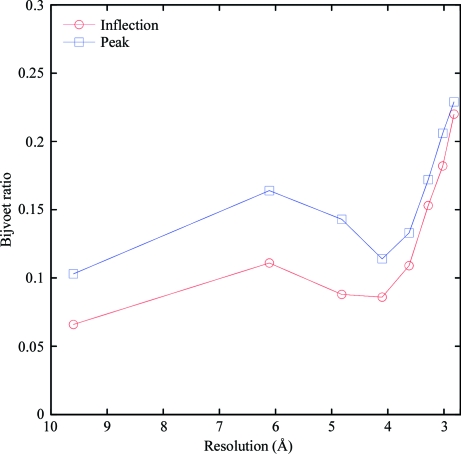
Graph of the Bijvoet ratios from the peak-wavelength and inflection-wavelength data from the GerE test case as a function of resolution. The peak wavelength is shown as blue squares and the inflection wavelength is shown as red circles.

Fig. 3 ▶ shows the fraction of the backbone built within 1 Å of the final deposited structure for each of these data sets for the current version of *CRANK* (version 1.4) *versus* the previous version (version 1.3). In total, 77 of 116 structures have greater than 60% of the structure built correctly; of these 77 structures, 66 are built to over 80% completeness. An example of an automatically built structure with a weak signal is GerE (Ducros *et al.*, 2001 ▶). The structure of GerE was originally solved with a four-wavelength selenomethionine MAD data set collected at 2.7 Å resolution and a native data set to 2.1 Å resolution. *CRANK* version 1.3 could build the structure using just the peak data set to a high degree, but failed to build the structure using just the SAD inflection data set. *CRANK* version 1.4 can build the structure to a high degree using either the peak or inflection data set. We are unaware of any other automated package or collection of algorithms that can build GerE using either the peak or inflection data set automatically. To give an indication of the anomalous signal, Fig. 4 ▶ plots the Bijvoet ratio (*i.e.* |Δ*F*|/|*F*|) as a function of resolution bin for the GerE peak and inflection data: the overall Bijvoet ratios for the peak and inflection data are 0.167 and 0.139, respectively.

For the 77 structures that were built automatically, sub­structure determination successfully terminated early in 69 of the cases. For 33 of the 69 cases the Luzzati parameter statistics in Bp3 allowed early termination, while in the remaining 36 cases the complete substructure was validated by an analysis of the *CRUNCH*2 statistics.

### 4.1. Analysis of data sets that were not automatically built

39 of the 116 data sets could not be built automatically by *CRANK*. 19 of the 39 data sets failed at substructure detection and could be built automatically if the resolution cutoff in *CRUNCH*2 was changed or if *SHELXC* and *SHELXD* were used in substructure detection. It should also be noted that the five cases that could not be built in version 1.4 but were successful in version 1.3 were all a consequence of the changes in the substructure-detection algorithm. These tests will be used to further debug and improve the development version of the multivariate |*F*_A_| calculation in *AFRO*.

For five of the 39 cases, *CRANK* in conjunction with a new SIRAS function for phasing leads to building when the current ‘uncorrelated’ function in *BP*3 had failed to produce an automatically traceable map. The multivariate SIRAS function for phasing will be released in the next version of *CRANK*.

The remaining 15 cases could not be built automatically or manually in *CRANK*. For seven of theses cases, Mueller-Dieckmann *et al.* (2007 ▶) had also failed to build the structures. Similarly, four other cases consisted of SAD experiments using derivative data sets from SIRAS experiments also containing a very weak signal. It is very likely that no currently available methods can build these structures and new methods need to be developed to build structures from such weak data. The remaining four cases that could not be built are from the JCSG repository: these structures can be built with currently available methods and the given data. The reasons why *CRANK* fails to build these data sets have yet to be determined.

## 5. Conclusions and future developments

Because of the new methods that we have developed, *CRANK* can build many more structures automatically and can build structures where current methods fail. *CRANK*’s robustness is shown by the large number of data sets that we use in this test that require very minimal input.

*CRANK*’s *ccp*4*i* interface is easy to use but does have some limitations: log files are only updated once a particular step in the pipeline has finished and users cannot manually stop a current step and proceed to a next step; the pipeline can only be terminated and the *CRANK* run must be restarted from the the beginning. Furthermore, although *CRANK* has an interface to *Coot* (Emsley *et al.*, 2010 ▶), it cannot show real-time updates of a model as a *CRANK* run proceeds. All of these shortcomings are being addressed and a new *PyQt* (http://www.riverbankcomputing.co.uk/software/pyqt/intro) interface for *CRANK* is currently being developed in collaboration with CCP4.

Although having an easy-to-use and powerful interface is important, the first priority for *CRANK* will always be the development of better methods to solve data sets that elude current methods. In the case of MAD data, current approaches in *CRANK* and elsewhere use univariate uncorrelated likelihood functions for *F*_A_ calculation, substructure phasing and the MLHL function for density modification and automated model building and refinement. Obviously, a multivariate MAD function could address the shortcomings in current approaches and could lead to structure solutions where current methods fail.

In the case of SAD data, the multivariate functions used in substructure phasing, density modification and model refinement only differ in the number of input variables and the parameterization. Although current algorithms separate these steps, the common mathematical framework suggests that all the information could be used simultaneously and combined optimally in a unified process using a single mathematical function, possibly resulting in substantial improvements.

## Appendix A Data sets

A total of 132 data sets were used and were composed of 78 data sets from the Joint Center for Structural Genomics (JCSG; http://www.jcsg.org/), 1vjn, 1vjr, 1vjz, 1vk4, 1vkm, 1vlm, 1vqr, 1z82, 1zy9, 1zyb, 2a2m, 2a2o, 2a3n, 2a6b, 2aml, 2avn, 2b8m, 2etd, 2etj, 2ets, 2etv, 2evr, 2f4p, 2fea, 2ffj, 2fg0, 2fg9, 2fna, 2fqp, 2fur, 2fzt, 2g0t, 2g42, 2gc9, 2nlv, 2nuj, 2nwv, 2o08, 2o1q, 2o2x, 2o2z, 2o3l, 2o62, 2o7t, 2o8q, 2obp, 2oc5, 2od5, 2od6, 2oh3, 2okc, 2okf, 2ooj, 2opk, 2osd, 2otm, 2ozg, 2ozj, 2p10, 2p4o, 2p7h, 2p7i, 2p97, 2pg3, 2pg4, 2pgc, 2pim, 2pn1, 2pnk, 2ppv, 2pr7, 2prr, 2prv, 2prx, 2pv4, 2pw4, 2b78 and 2b79; 23 data sets from Mueller-Dieckmann *et al.* (2007 ▶), 2g4h, 2g4i, 2g4j, 2g4k, 2g4p, 2g4q, 2g4l, 2g4n, 2g4o, 2g4r, 2g4s, 2g4t, 2g4u, 2g4v, 2g4w, 2g4x, 2g4y, 2g4z, 2ill, 2g51, 2g52, 2g54 and 2g55; and 31 from various other individual data-set contributors, 1e42 (Owen, Vallis *et al.*, 2000 ▶), 1e6i (Owen, Ornaghi *et al.*, 2000 ▶), 1hf8 (Ford *et al.*, 2001 ▶), 2ahy (Shi *et al.*, 2006 ▶), 2hba (J.-H. Cho, S. Sato, E. Y. Kim, H Schindelin & D. P. Raleigh, unpublished work), 2o0h (Sun *et al.*, 2007 ▶), 2rkk (Xiao *et al.*, 2008 ▶), 3bpj (L. Nedyalkova, B. Hong, W. Tempel, F. Mac­Kenzie, C. H. Arrowsmith, A. M. Edwards, J. Weigelt, A. Bochkarev & H. Park, unpublished work), 2fdn (Dauter *et al.*, 1997 ▶), 1of3 (Boraston *et al.*, 2003 ▶), 1i4u (Gordon *et al.*, 2001 ▶), 1dw9 (Walsh *et al.*, 2000 ▶), 1v0o (Holton *et al.*, 2003 ▶), 1fse (Ducros *et al.*, 2001 ▶), 1xib (Carrell *et al.*, 1989 ▶), 1fj2 (Devedjiev *et al.*, 2000 ▶), 1h29 (Matias *et al.*, 2002 ▶), 1c8u (Jia *et al.*, 2000 ▶), 1lvy (Schiltz *et al.*, 1997 ▶), 1lz8 (Dauter *et al.*, 1999 ▶), 1e3m (Lamers *et al.*, 2000 ▶), 1ga1 (Dauter *et al.*, 2001 ▶), 1djl (White *et al.*, 2000 ▶), 1dtx (Skarzynski, 1992 ▶), 1dpx (Weiss, 2001 ▶), 1mso (Smith *et al.*, 2003 ▶), 1ocy (Thomassen *et al.*, 2003 ▶), 1rju (Calderone, 2004 ▶), 1rgg (Sevcik *et al.*, 1996 ▶), 1m32 (Chen *et al.*, 2002 ▶) and a subtilisin data set (Betzel *et al.*, 1988 ▶; Dauter *et al.*, 2002 ▶). Data where a program terminated abnormally in either pipeline were excluded from the statistics and graphs presented, resulting in 116 data sets.
